# Erratum: Stella, G.M. et al. Brain Metastases from Lung Cancer: Is MET an Actionable Target? *Cancers* 2019, *11*, 271

**DOI:** 10.3390/cancers11050644

**Published:** 2019-05-10

**Authors:** Giulia M. Stella, Alessandra Corino, Giulia Berzero, Stefan Kolling, Andrea R. Filippi, Silvia Benvenuti

**Affiliations:** 1 Department of Medical Sciences and Infectious Diseases, Unit of Respiratory System Diseases, IRCCS Fondazione Policlinico San Matteo, 27100 Pavia, Italy; corinoalessandra@gmail.com (A.C.); stefan.kolling01@universitadipavia.it (S.K.); 2 IRCCS Istituto Casimiro Mondino, 27100 Pavia, Italy; giulia.berzero@mondino.it; 3 Department of Medical Sciences and Infectious Diseases, Unit of Radiation Therapy, IRCCS Fondazione Policlinico San Matteo, 27100 Pavia, Italy; a.filippi@smatteo.pv.it; 4 Candiolo Cancer Institute, FPO-IRCCS, 10060 Candiolo TO, Italy; silvia.benvenuti@ircc.it

The authors wish to make the following corrections to this paper [1]:

The affiliation of the last author Silvia Benvenuti should be “Candiolo Cancer Institute, FPO-IRCCS, 10060 Candiolo TO, Italy”.

The Funding section should be changed to: Ricerca Corrente—IRCCS Fondazione Policlinico San Matteo to G.M.S. and Italian Ministry of Health Ricerca Corrente 2019 to Candiolo Cancer Institute, FPO-IRCCS, Candiolo (TO) Italy.

These changes do not affect the scientific results. The manuscript will be updated and the original version will remain online on the article webpage, with a reference to this erratum. The authors would like to apologize for any inconvenience caused to the readers by this change.
